# Supplementary material for: Volatilized Ammonia Supports Subterranean Ecosystems with Unusual Nitrogen Isotopic Signatures
Source: Environ Sci Technol. 2026 May 15;60(21):15068–77. doi: 10.1021/acs.est.6c00328 (PMC13235542; doi:10.1021/acs.est.6c00328)
Supplement: Supplementary file 1 [file es6c00328_si_001.pdf]

Supplementary Figures for:

Volatilized ammonia supports subterranean ecosystems with unusual nitrogen isotopic signatures

Mackenzie B. Best[a*#], Scott D. Wankel[b], Heather V. Graham[c], Jennifer C. Stern[c], Jennifer Macalady[d], Maurizio Mainiero[e,f], Stefano Recanatini[f], Sandro Mariani[g], Ilenia D'Angeli[h], Ilaria Vaccarelli[i], Daniel S. Jones[a,j*]

[a]Department of Earth and Environmental Sciences, New Mexico Institute of Mining and Technology, Socorro, NM 87801 USA
[b]Department of Marine Chemistry and Geochemistry, Woods Hole Oceanographic Institution, Woods Hole, MA 02543 USA
[c]NASA Goddard Space Flight Center, Greenbelt, MD 20771 USA
[d]Department of Geosciences, The Pennsylvania State University, State College, PA 16802 USA
[e]Federazione Speleologica Marchigiana, Jesi, Marche, 60035 Italy
[f]Gruppo Speleologico Marchigiano, Ancona, Marche, 60100 Italy
[g]Gruppo Speleologico CAI Fabriano, Fabriano, 60044 Italy
[h]Italian Institute of Speleology, Bologna, Emilia-Romagna, 40138 Italy
[i]Water Research Institute, National Research Council, Verbania, 28922 Italy
[j]National Cave and Karst Research Institute, Carlsbad, NM 88220 USA

[*]Email: mbbest@sandia.gov
[*]Email: daniel.s.jones@nmt.edu

Present Addresses:
[#]M.B.B.: Environmental Systems Biology, Sandia National Laboratories, Albuquerque, NM 87123 USA

This document (6 pages) contains Supplementary Figures S1-S5 Supplementary Tables S1-S5 are included as a separate document.

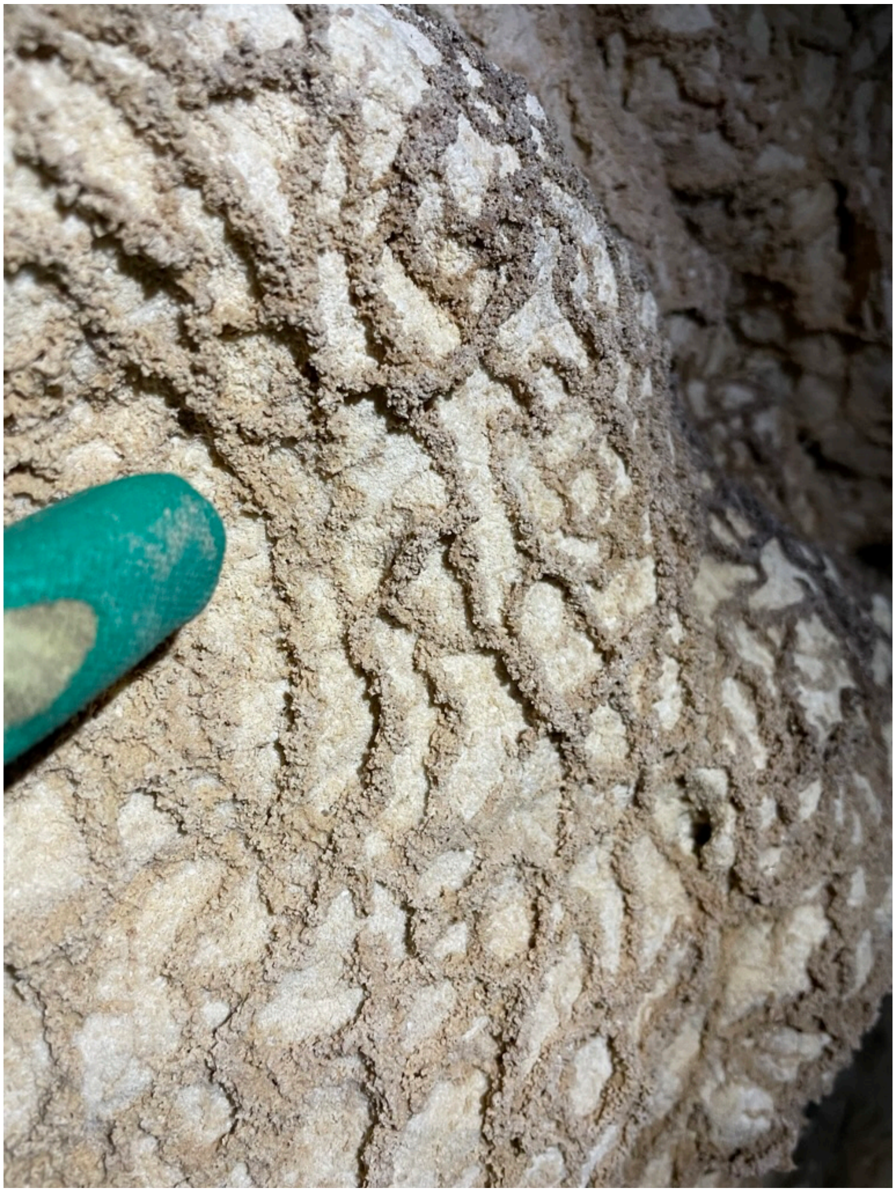

Supplementary Figure S1. Biovermiculations in Grotta Bella.

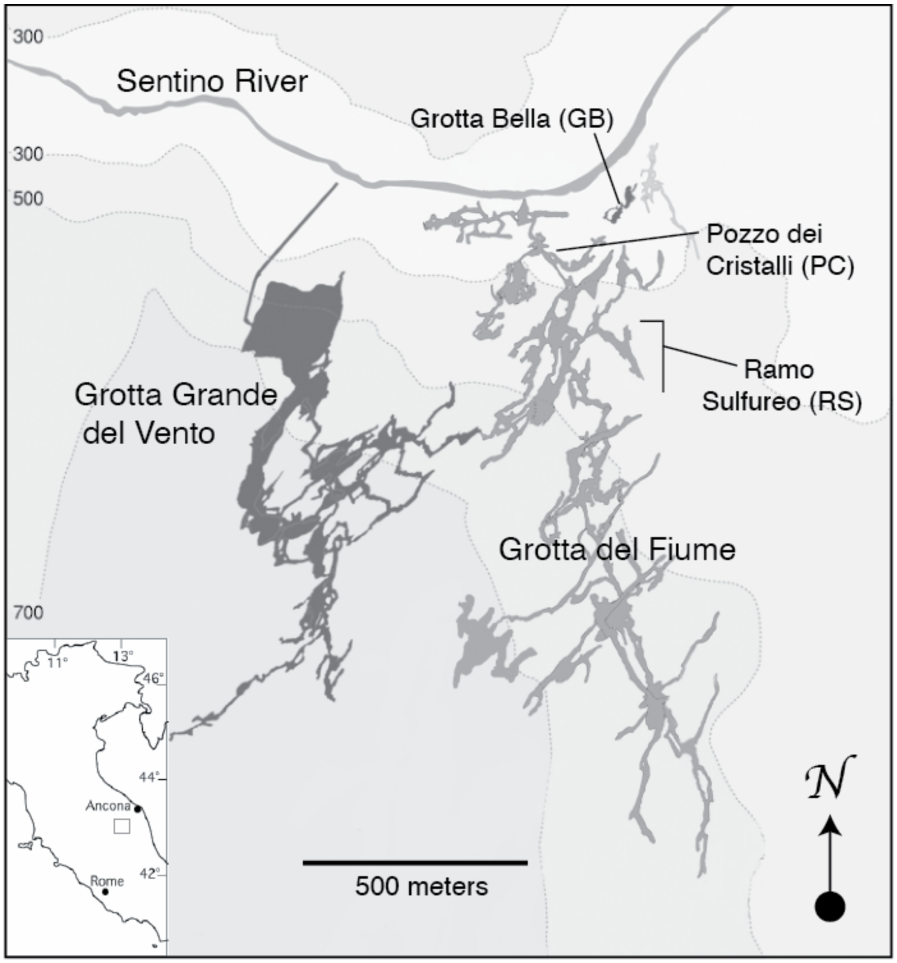

Supplementary Figure S2. Map of the Frasassi cave system, showing the three sampling locations used in this study. Base map courtesy of the Gruppo Speleologico CAI di Fabriano.

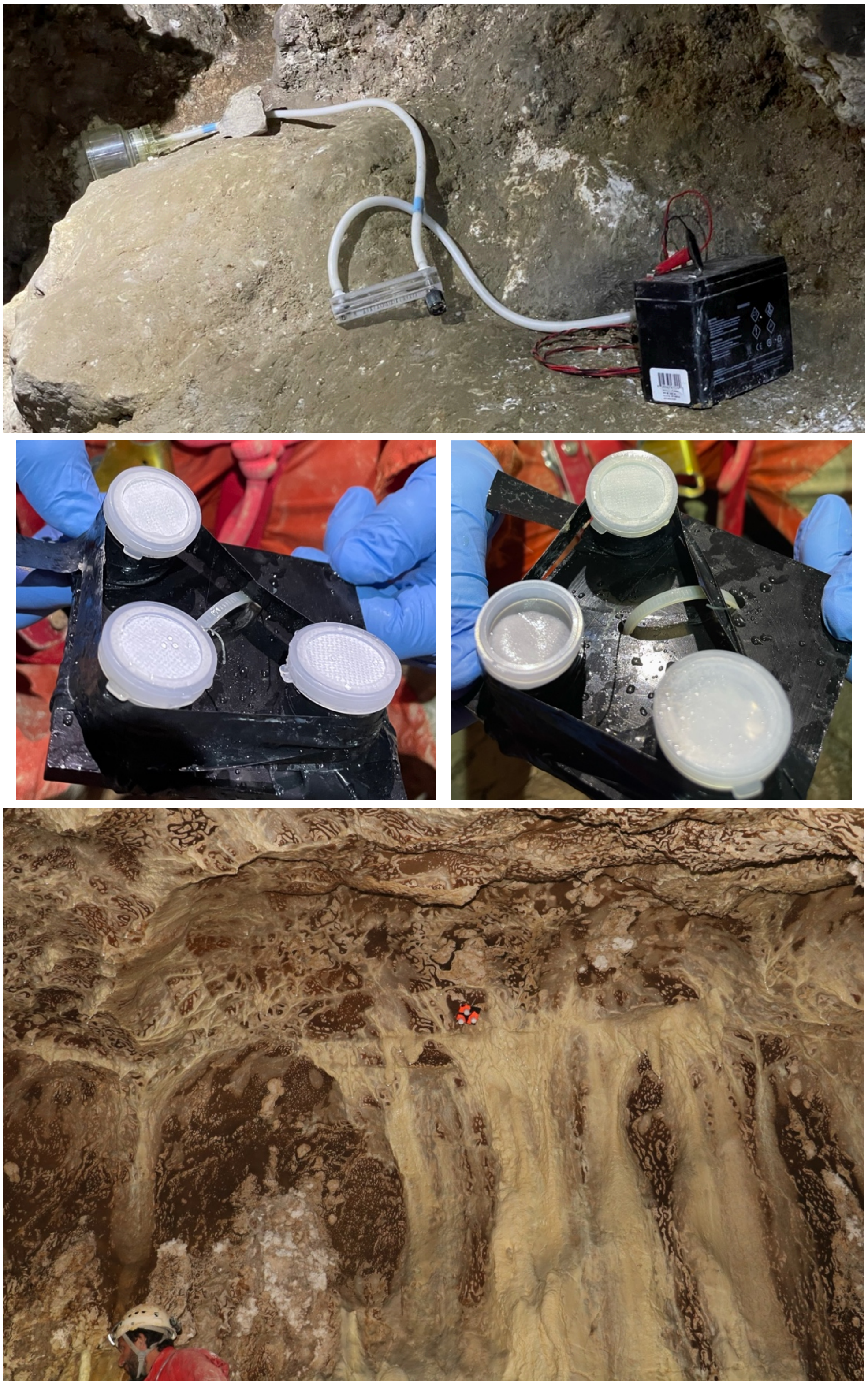

Supplementary Figure S3. Active (top) and passive (bottom three images) cave air samplers. In the passive samplers, the acid-treated filter is protected by a gas-permeable cover; image in the middle right shows one that was wet upon collection and the bound ammonium likely lost. The bottom image shows a deployed passive ammonia sampler.

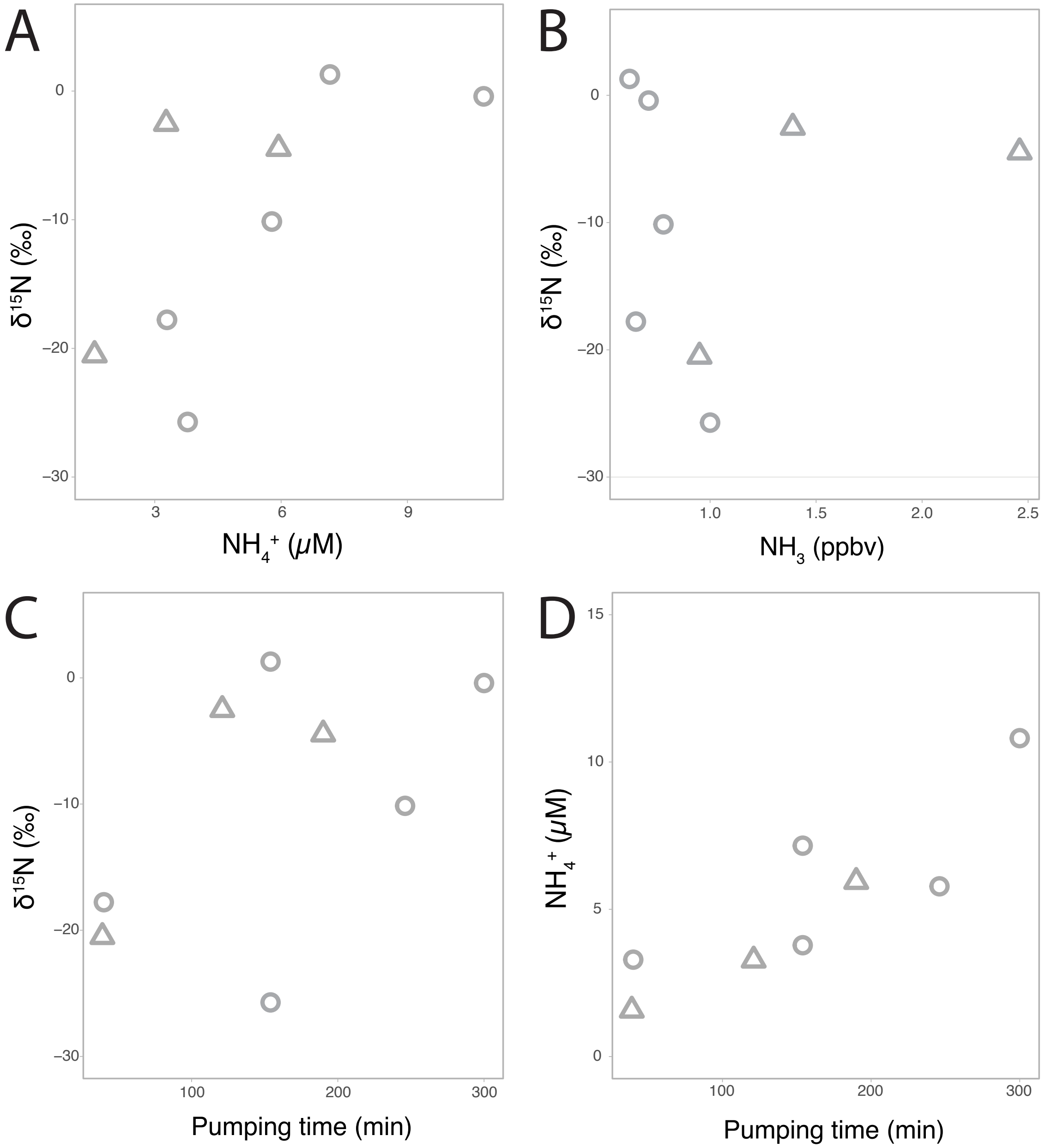

Supplementary Figure S4. $\delta^{15}N$ of ammonium recovered from active cave air samplers versus (A) the concentration of ammonium concentration eluted from filters, (B) the calculated concentration of NH_3(g) in the cave air, and (C) pumping time. Panel D shows the concentration of ammonium concentration eluted from filters versus pumping time. Circles indicate samples from site GB, and triangles indicate samples from site PC.

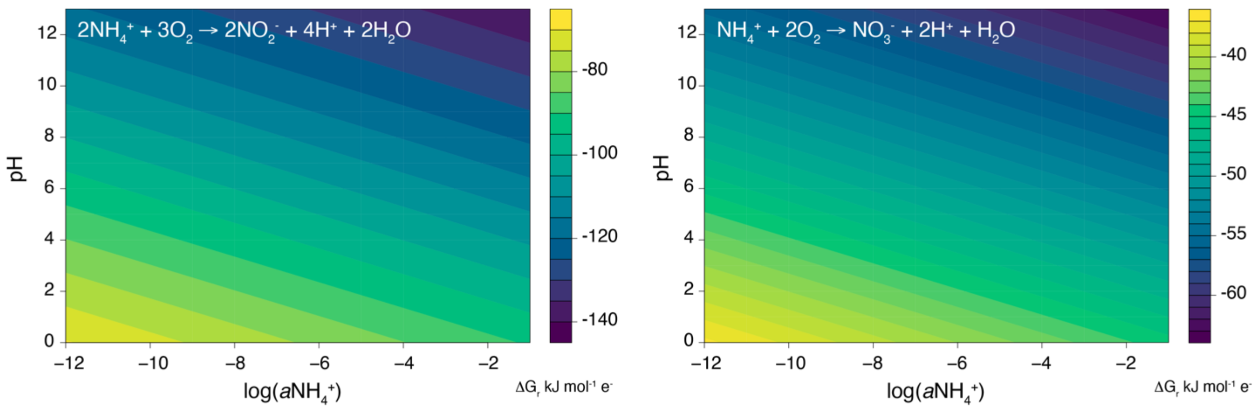

Supplementary Figure S5. Gibbs free energy of ammonium oxidation to nitrite and nitrate as a function of pH and ammonium concentration, assuming a temperature of 25°C, 20.9% oxygen, and activities of nitrate and nitrite of 10^{-12}.
